# Supplementary material for: Foot arch rigidity in walking: In vivo evidence for the contribution of metatarsophalangeal joint dorsiflexion
Source: PLoS One. 2022 Sep 8;17(9):e0274141. doi: 10.1371/journal.pone.0274141 (PMC9455856; doi:10.1371/journal.pone.0274141)
Supplement: S1 Appendix — (DOCX) [file pone.0274141.s001.docx]

| **Table 1.** **List of marker and joint names and locations.** | |
| --- | --- |
| *Marker* | *Landmark* |
| C | Approximate location of Achilles tendon insertion |
| PT | Lateral aspect of peroneal tubercle |
| ST | Medial aspect of sustentaculum tali |
| N | Medial aspect of navicular tuberosity |
| MB5 | Lateral aspect of the base of the 5th metatarsal |
| MH1 | Medial aspect of head of the 1st metatarsal |
| MH5 | Lateral aspect of head of the 5th metatarsal |
| T1 | Just distal to 1st metatarsophalangeal joint |
| T2 | Between proximal and distal interphalangeal joints of 2nd toe |
| T5 | Between proximal and distal interphalangeal joints of 5th toe |
| Midtarsal joint | Midpoint(N, MB5) |
| MTP joint | Orthogonal projection of MH1 to T2 onto MH1 to MH5 (transverse plane) |

| **Table 2. Local segment frame definitions.** | | |
| --- | --- | --- |
| *Segment* | *Long Axis (begins at segment origin)* | *Plane* |
| Rearfoot | C to midpoint(ST, PT) | C, ST, PT |
| Forefoot | Midtarsal joint to MTP joint | Midtarsal joint, MH1, MH5 |
| Toe | MTP joint to T2 | T1, T2, T5 |
| C = calcaneus; ST = sustentaculum tali; PT = peroneal tubercle; MTP = metatarsophalangeal; MH1 = metatarsal head; T = toe. | | |

The toe segment of the foot model incorporated all five toes, with the origin defined using the projection of the vector from MH1 to T2 in the transverse plane onto the vector from MH1 to MH5 in the transverse plane. The forefoot segment spanned from the midpoint between the navicular tuberosity and the base of the fifth metatarsal to the toe segment origin. The rearfoot segment encompassed foot structures distal to the malleoli and posterior to the navicular tuberosity and fifth metatarsal base. Foot segment masses were estimated using foot mass as a proportion of body mass from Dempster [1]. Foot segment volume and moments of inertia were found by modeling the rearfoot as a cylinder and the forefoot and toes as elliptical cylinders [2]. Each foot segment’s mass was estimated based on its proportion of total foot volume and an assumption of constant density [2]. Joining the rearfoot and the shank segment was the ankle joint, whose joint center was coincident with the midpoint between the medial and lateral malleolus. The shank segment was defined as in the ISB standard [3].

**References**

1. Dempster W. Space requirements of the seated operator. Ohio: Wright-Patterson Air Force Base; 1955. Report No.: WADC technical report 55-129.
2. Bruening DA, Cooney KM, Buczek FL. Analysis of a kinetic multi-segment foot model. Part I: Model repeatability and kinematic validity. Gait Posture. 2012;35(4): 529–534. doi:10.1016/j.gaitpost.2011.10.363.
3. Wu G, Siegler S, Allard P, Kirtley C, Leardini A, Rosenbaum D, et al. ISB recommendation on definitions of joint coordinate system of various joints for the reporting of human joint motion—part I: ankle, hip, and spine. J Biomech. 2002;35: 543–548. doi:10.1016/S0021-9290(01)00222-6.
