# Supplementary figures and images for: Foot arch rigidity in walking: In vivo evidence for the contribution of metatarsophalangeal joint dorsiflexion
Source: PLoS One. 2022 Sep 8;17(9):e0274141. doi: 10.1371/journal.pone.0274141 (PMC9455856; doi:10.1371/journal.pone.0274141)

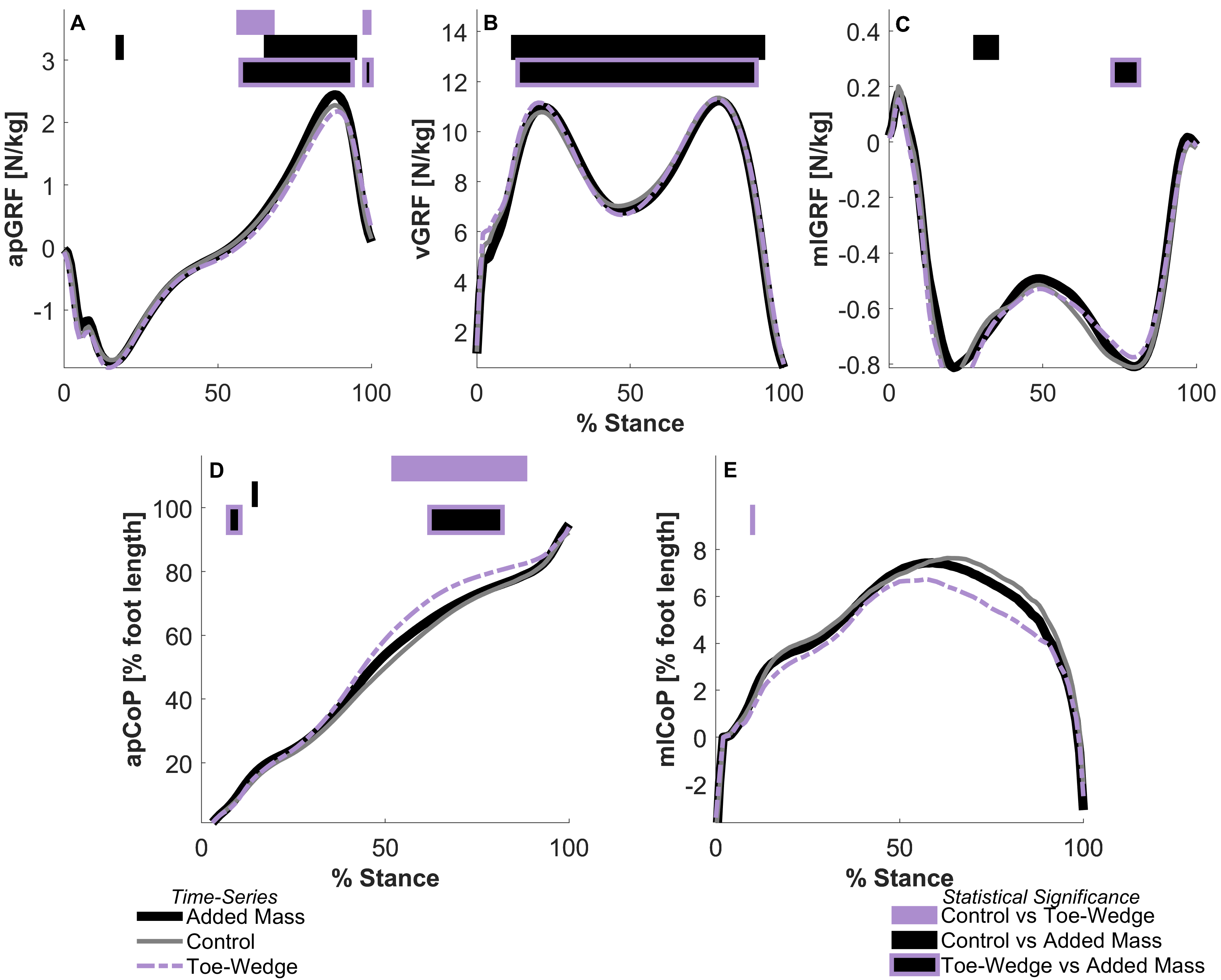

Supplement: S1 Fig — Panels A-C: Anterior-posterior (ap), vertical (v), and medial-lateral (ml) ground reaction force profiles (GRFs) normalized to body mass in all three planes in the added mass (AM), control, and toe-wedge (TW) conditions. Panels D-E: Anterior-posterior and medial-lateral center of pressure (CoP) profiles normalized to foot length in the added mass, control, and toe-wedge conditions. Horizontal bars top of the left panel indicate the timing of a statistically significant time-series differences between the control and toe-wedge conditions using a two-tailed paired t-test (α = 0.017 after Bonferroni correction). The top (purple) horizontal bar indicates a difference between control and toe-wedge conditions, the middle (black) bar represents a difference between the control and added mass conditions, and the bottom (black with purple outline) bar denotes a difference between the toe-wedge and added mass conditions. (TIF) [file pone.0274141.s002.tif]
