# Supplementary material for: Foot arch rigidity in walking: In vivo evidence for the contribution of metatarsophalangeal joint dorsiflexion
Source: PLoS One. 2022 Sep 8;17(9):e0274141. doi: 10.1371/journal.pone.0274141 (PMC9455856; doi:10.1371/journal.pone.0274141)
Supplement: S1 Table — Values represent mean ± standard deviation and are normalized to total mass in each condition. (DOCX) [file pone.0274141.s003.docx]

**Table S1. Impulse values determined from ground reaction force-time profiles.**

|  | *Condition* | | |
| --- | --- | --- | --- |
| *Component* | Control  [Ns/kg] | Toe-Wedge  [Ns/kg] | Added Mass  [Ns/kg] |
| Anterior-posterior† | 0.03 ± 0.08 | -0.01 ± 0.05 | 0.05 ± 0.07 |
| Vertical | 4.99 ± 0.22 | 4.99 ± 0.22 | 5.00 ± 0.24 |
| Medial-lateral | -0.32 ± 0.07 | -0.33 ± 0.08 | -0.32 ± 0.06 |
| Values represent mean ± standard deviation and are normalized to total mass in each condition.  †indicates a difference between the toe-wedge and added mass conditions (α = 0.017). | | | |
