# Supplementary material for: Foot arch rigidity in walking: In vivo evidence for the contribution of metatarsophalangeal joint dorsiflexion
Source: PLoS One. 2022 Sep 8;17(9):e0274141. doi: 10.1371/journal.pone.0274141 (PMC9455856; doi:10.1371/journal.pone.0274141)
Supplement: S2 Table — Values represent mean ± standard deviation and are normalized to the product of body mass, acceleration due to gravity, and leg length. (DOCX) [file pone.0274141.s004.docx]

| **Table S2. Dimensionless joint mechanical work values.** |
| --- |
| \|  \|  \| *Condition* \| \| \| \| --- \| --- \| --- \| --- \| --- \| \| *Joint* \| *Component* \| Control  [$\times$ 10^-2^] \| Toe-Wedge  [$\times$ 10^-2^] \| Added Mass  [$\times$ 10^-2^] \| \| MTP joint \| Negative^#^† \| -1.73 ± 0.50 \| -1.87 ± 0.74 \| -2.22 ± 0.79 \| \| Positive \| 0.44 ± 0.10 \| 0.51 ± 0.12 \| 0.47 ± 0.08 \| \| Midtarsal joint \| Negative^#^ \| -0.72 ± 0.13 \| -0.81 ± 0.23 \| -0.88 ± 0.27 \| \| Positive^#^† \| 1.78± 0.31 \| 1.75 ± 0.38 \| 2.22 ± 0.38 \| \| Ankle joint \| Negative \| -0.11 ± 0.31 \| -0.15 ± 0.36 \| -0.12 ± 0.32 \| \| Positive^#^† \| 2.20 ± 0.38 \| 2.33 ± 0.40 \| 2.74 ± 0.45 \| |
| Values represent mean ± standard deviation and are normalized to the product of body mass, acceleration due to gravity, and leg length.  ^#^ indicates a difference between the added mass and control conditions (α = 0.017).  †indicates a difference between the toe-wedge and added mass conditions (α = 0.017). |
